# Perspective: Raman spectroscopy for detection and management of diseases affecting the nervous system

**DOI:** 10.3389/fvets.2024.1468326

**Published:** 2024-10-21

**Authors:** John L. Robertson, Amr Sayed Issa, Ryan S. Senger

**Affiliations:** ^1^Department of Biomedical Engineering, Virginia Tech, Blacksburg, VA, United States; ^2^Rametrix Technologies, Inc., Blacksburg, VA, United States; ^3^Veterinary and Comparative Neurooncology Laboratory, Virginia Tech, Blacksburg, VA, United States; ^4^Department of Biological Systems Engineering, Virginia Tech, Blacksburg, VA, United States

**Keywords:** Raman, spectroscopy, urinalysis, glioma, brain, tumors

## Abstract

Raman spectroscopy (RS) is used increasingly for disease detection, including diseases of the nervous system (CNS). This Perspective presents RS basics and how it has been applied to disease detection. Research that focused on using a novel Raman-based technology—Rametrix^®^ Molecular Urinalysis (RMU)—for systemic disease detection is presented, demonstrated by an example of how the RS/RMU technology could be used for detection and management of diseases of the CNS in companion animals.

## Introduction

The timely and accurate detection of disease affecting the central nervous system (CNS) is key to initiating and providing care for symptomatic patients. Currently, detection of CNS disease relies on data from an adequate medical history, physical and neurologic examinations, imaging and laboratory studies, and response, or lack of response, to treatment. Many laboratory tests rely on the analysis of blood, plasma, serum, and cerebrospinal fluid (CSF) for detection of injury or quantifying organ dysfunction that may indirectly affect CNS activity. Changes in markers of inflammation, such as white blood cell counts, may indicate injury to the structure of the CNS, the presence of infection, or septicemia. These common laboratory procedures are relatively imprecise for localizing disease processes to the CNS or for early detection of disease. While repeated laboratory measurements may change during treatment of disease, they are insensitive in assessing treatment efficacy or rarely used to manage treatment and can be slow to detect outcomes. Urinalysis has not been used commonly in the detection and management of CNS disease, due to the perception that it provides only minimal information reflecting either CNS health or dysfunction.

There is intense interest in human medicine for discovering and using biomarkers of CNS disease. According to the food and drug administration (FDA), a biomarker is “a defined characteristic that is measured as an indicator of normal biological processes, pathogenic processes, or biological responses to an exposure or interventions.” This definition was expanded by the National Academy of Science, Engineering, and Mathematics to recognize that biomarkers should demonstrate a response to therapeutic interventions or environmental exposures (1). Much of the research in CNS biomarkers has focused on detection of human neurodegenerative diseases, including forms of dementia, Parkinson’s disease, Huntington’s disease, or traumatic brain injury (2–8). Biomarkers of human CNS disease have been used to define the appearance, severity, and progression of disease, and can be a valuable tool in development of drugs for amelioration of disease (1). It is well-recognized that the presentation and progression of disease is patient-specific and that biomarkers may not be useful for the detection and management of disease in all patients. Likewise, lengthy longitudinal studies are frequently needed to demonstrate that specific biomarkers accurately define the trajectory of disease and treatment responses. The cost of developing biomarkers for human CNS disease detection/management is very high—tens of millions of dollars. Recent advances in the development of blood and/or CSF biomarkers suggest they will move from research laboratory studies to clinical use within the next decade, overcoming current limitations of accuracy and cost.

In perspective: research and clinical use of CNS disease biomarkers for animal patient management lags significantly behind research/use for human patient management. Economic and technological considerations will continue to limit development of biomarkers specifically for clinical use in animals, although the use of translational animal models may provide opportunities to apply “human biomarkers” as surrogate “animal biomarkers.”

Given the limitations of current standard clinical laboratory testing, and lack of availability of relevant biomarkers for animal CNS disease, there is a need for the development and/or use of non-invasive or minimally invasive, accessible, and affordable technologies for improving patient management and outcomes. In perspective: Raman spectroscopy (RS) of biological samples may meet this need.

## Raman spectroscopy basics

RS is a mature, well-studied, and powerful technology that has been applied to analysis of the chemical composition of a wide variety of solids and liquids, including biological specimens (9–18). The analysis can be performed quickly (samples scanned in seconds) on either solids or liquids. Sample volumes required for analysis are small—1 mL of liquids, a few milligrams of most solids—and samples do not need to be chemically-modified for analysis. Raman spectrometers are readily available from commercial sources, are very durable (lifecycle of years is common), have a small footprint (cell-phone size), inexpensive ($5 K–25 K), and operate in ambient conditions typical in medical clinics.

**Figure 1:**
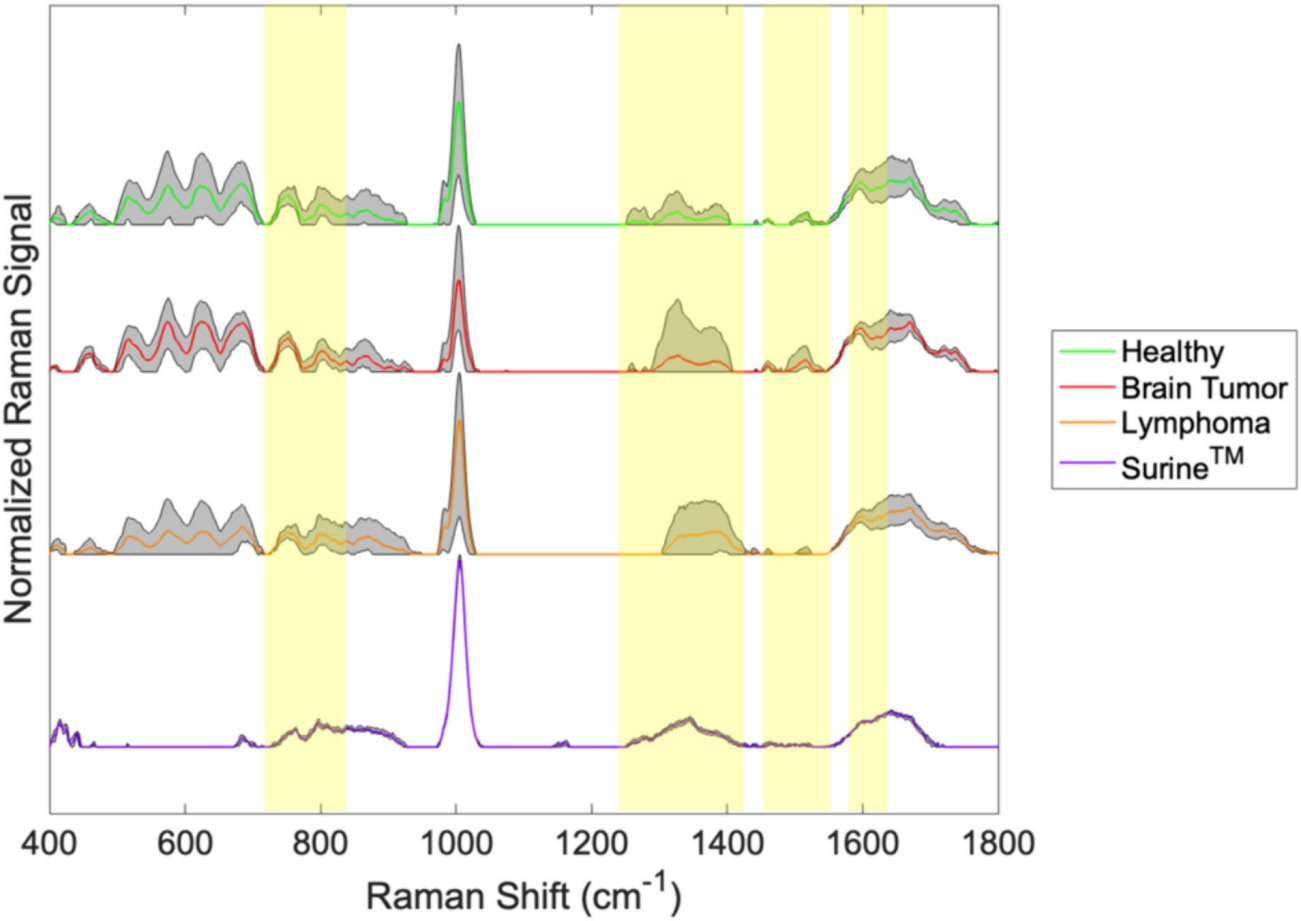
Spectral fingerprints for canine urine produced from ISREA baselining and processing raw Raman spectral data.

Irradiation of molecular mixtures (e.g., biological fluids like urine or foodstuffs) with wavelength-specific laser energy, produces weak polarization shifts from deformation/relaxation of chemical bonds in hundreds of distinct molecules in the specimens. Bond chemistries, as opposed to individual molecules, are resolved on resulting spectra; however, these are often linked back to biological molecules (e.g., amino acids, carbohydrates, etc.) using spectral libraries (9, 10). To demonstrate the distinguishing capabilities of RS, representative Raman spectra of urine specimens and the urinalysis standard Surine™ from our recent canine study (19) are shown in Figure 1. These were produced from urine samples from healthy dogs and from dogs with brain tumors (including meningioma, glioblastoma, astrocytoma, or oligodendroglioma) or lymphoma. Colored lines are average spectra from all samples of that type collected. Gray shaded regions represent the range observed at each wavenumber. Yellow highlighted regions demonstrate where differences in the spectra were recognizable by the naked eye. It is easy to see that these samples all share a common base level chemical composition (i.e., the unshaded regions), with important spectral differences (i.e., yellow highlighted regions). Thus, a Raman spectrum can be used to not only determine the broader type of fluid (i.e., analytical standard or urine) but more detailed information (i.e., the changing urine metabolome). Without knowing the exact chemicals that give rise to the differentiating peaks in the Raman spectra, we can treat each spectrum as a spectral “fingerprint” of that sample. Analysis of several different types of samples (i.e., urine samples from different patients) can give rise to a large spectral library. This becomes useful when one wishes to know if a disease/metabolic condition is present. Multivariate statistical methods are used to match the Raman spectrum of the new unknown sample to the sample it most closely resembles in the database. We used this strategy in several case studies (19–31).

### RS chemometric analysis

The differences between the samples in Figure 1 are clear in some cases (e.g., between 1,200 and 1,425 cm^−1^), but this is not always the case. For example, the differences between the spectra of brain tumor patients and healthy dogs are difficult to discern around 800 cm^−1^ in Figure 1. Often, computer algorithms are needed to discover key differences in spectral fingerprints in a procedure called “chemometric analysis.” Given that the Raman spectra of other complex biological fluids, such as plasma, serum, whole blood, and cerebrospinal fluid (10, 11, 18, 31–35) are composed of literally thousands of peaks and valleys (at points designated as wavenumbers), the chemometric fingerprinting approach is likely to be effective with these.

**Figure 2:**
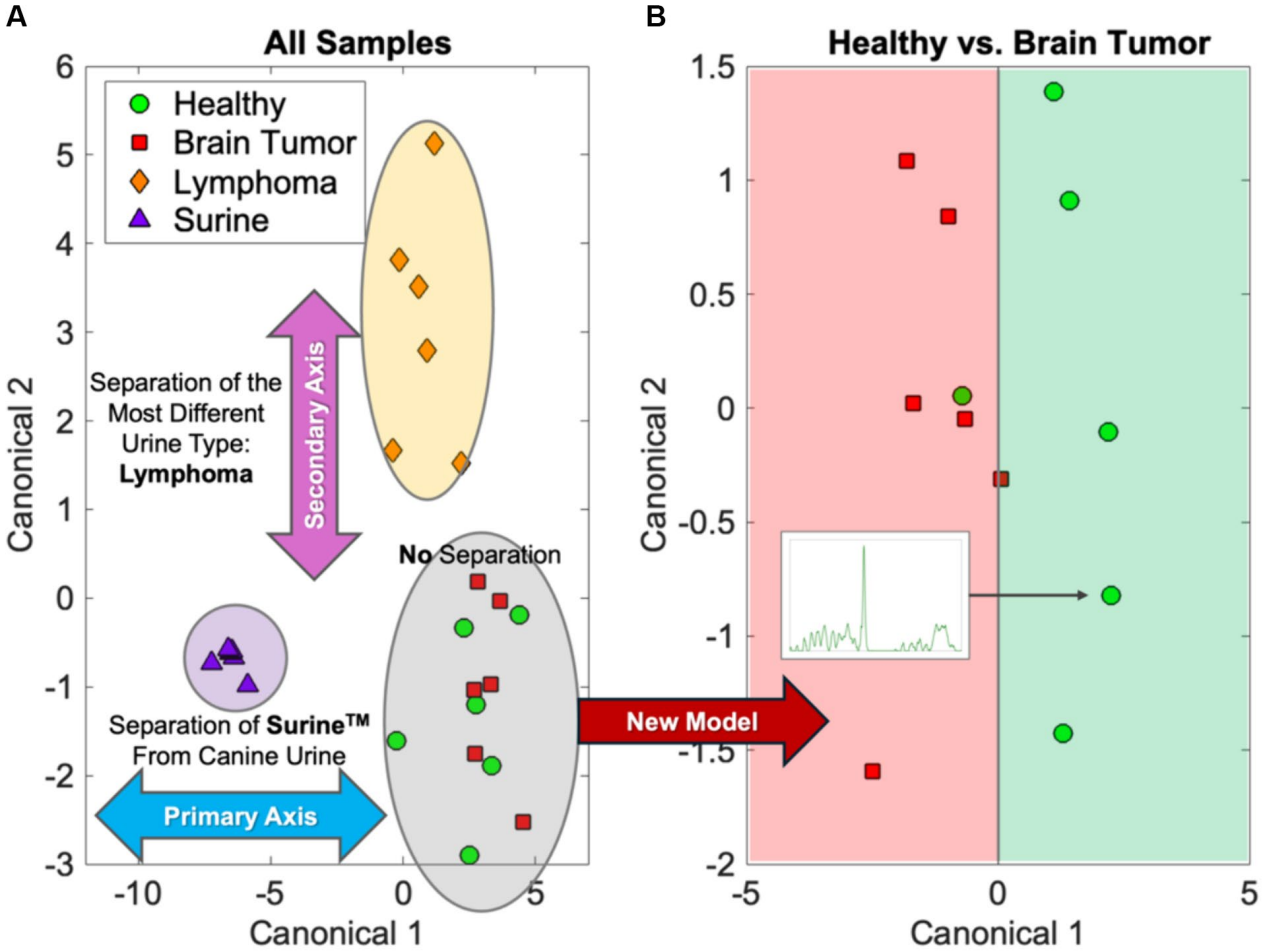
MANOVA modeling of the urine fingerprints in Figure 1. **(A)** The initial separations of lymphoma and Surine samples and **(B)** separation of the more similar healthy and brain tumor samples.

Chemometric comparisons of spectral fingerprints can be done by a wide variety of statistical, machine learning (ML), and artificial intelligence (AI) models (36–40). Multivariate analysis of variance (MANOVA) modeling of urine fingerprints in Figure 1 is shown in Figure 2A. MANOVA, a supervised modeling technique, seeks differences between groups of spectra and exploits those to create clusters. The process of MANOVA model-building is referred to as “training,” and these results are shown in Figure 2. Supervised models require “testing,” where spectra unknown to the model are processed and model predictions are compared with known results. For urine screening, the accuracy, sensitivity, specificity, positive-predictive value (PPV), and negative-predictive value (NPV) are often used to describe model performance (41). Much can be learned from MANOVA model training. In Figure 2A, Raman spectra of Surine™ were separated along Canonical 1, the primary MANOVA axis. This was the “easiest” separation, consistent when comparing Surine™ urine control spectra to the other urine spectra in Figure 1. Lymphoma urine spectra were separated along with second MANOVA axis (Canonical 2), leaving spectra from healthy dogs and dogs with brain tumors clustered together. Thus, urine of lymphoma patients is relatively easy to identify by the model. This left urine from healthy dogs and those with brain tumors clustered together. Separating these required a new model, where only these two groups were included. The MANOVA training model is shown in Figure 2B. However, when tested and validated with leave-one-out cross validation, accuracy >90% was obtained, with 100% sensitivity and >83% specificity for identifying a urine sample from a dog with brain tumor. We used a small number of samples in each group for this analysis, as a preliminary proof-of-concept - that needs more validation with larger datasets. However, we show how multiple MANOVA models can be used to separate out and classify very different samples first and then dedicate models to focus on samples that have few differences from one another.

In perspective: As databases of Raman spectra of biological molecules improve in breadth and availability, our ability to cross-reference more regions of the spectral fingerprint and confirm analytical results with mined literature data will improve. The procedure of spectral fingerprint reduction with computational and statistical techniques also offers the opportunity to identify new molecular contributors and specimen metabolomic differences due to the presence of a disease. These new metabolomic markers can be verified through other analytical means to further validate the fingerprint.

### Medical applications of RS, with special reference to CNS disease

The value of RS in detection and management of human diseases has become well-recognized in the past decade, in studies ranging from cancer detection (18, 42–48), to cardiovascular dysfunction (49), and as an aid to theranostics (50). The interested reader is encouraged to delve into the substantial medical literature on uses of RS for disease detection and management.

There has also been substantial interest in using RS for detection of CNS disease. Ranasinghe et al. (3, 4) published a comprehensive review of the uses of RS and “liquid biopsy” techniques for studying human brain disorders, specifically neurodegenerative diseases such as Alzheimer’s, Parkinson’s, and Huntington’s disease(s), amyotrophic lateral sclerosis, and traumatic brain injury (TBI), using blood, plasma, CSF, tears, and saliva samples. This excellent review also discussed chemical- and nanoparticle-RS signal enhancement techniques for improving detection of small molecules in biological fluids. Limitations in RS analysis/technology, including problems associated with reproducibility, sample composition (mixing) variation, quantification of molecular species in complex biological fluids like blood and CSF, interfering chemical and physical phenomena, and data analysis are reviewed in their work. Chen et al. (6) similarly reviewed the uses of RS for detection of neurodegenerative diseases, while also discussing the societal burden posed by these diseases in aging human populations. Terrones et al. (5) discussed the use of RS and tagged tracer molecules for studying the pathogenesis of brain disorders (neurodegenerative disease, TBI/ischemia, and CNS neoplasia), as well as for assessing the localization and kinetics of facilitated drug delivery. In perspective: while this work in human patients demonstrates that the applicability of RS to the study of brain disorders, it is limited to clinical research applications that are not directly applicable to veterinary neurology, given profound differences in the spectrum of spontaneous disease between human and companion animals.

Recently, RS has been used to detect and classify brain neoplasms (42, 51). Novel sensors, imaging modalities, and surface enhanced RS (SERS) for the detection and management of gliomas were reviewed by Thenuwara et al. (52). Several studies reported intriguing results of scanning of brain neoplasm biopsies or serum from patients with tumors. Riva et al. (53) scanned fresh (not fixed or frozen) biopsies from 63 patients undergoing surgical resection of gliomas. Using RS and ML, they were able to discriminate between healthy, non-neoplastic tissue and tumor tissue with an accuracy of 82% and noted that 19 unique Raman “bands” were the basis for discrimination. They, and others (51, 54–56), suggested that RS probes could be used intra-operatively to define tumor margins during resection—always a significant challenge with gliomas. Zhang et al. (57), using RS, compared serum sample from healthy individuals (*n* = 86) and patients with glioma [high-grade glioma (HGG) *n* = 75, low-grade glioma (LGG) *n* = 60]. They were able to classify, based on differences in serum Raman spectra, differences between healthy, HGG, and LGG groups with an accuracy of 94.12%. In perspective: these studies clearly have application to the practice of veterinary neurology and neurosurgery.

### Molecular urinalysis

Urine is a readily available complex fluid that can easily be obtained non-invasively or with minimal invasion (cystocentesis, catheterization) in companion animals. It is the product of systemic physiologic and pathologic processes, metabolism, and renal function—in essence, a dynamic “liquid biopsy” of the body. Studies have shown that normal human urine contains more than 2,500 separate chemical entities, many of them present in micromolar concentrations (11).

The composition and physical properties of urine in healthy people and animals varies widely each day. Changes in urine volume and urea content, for example, are predictably related to many physiologic/metabolic factors including the state of hydration, water intake, physical activity, and diet. The presence of miniscule amounts of hemoglobin/erythrocytes, sloughed genitourinary tract cells, and flora/fauna from the lower urinary tract is recognized and accepted as part of normal urine composition in free-catch urine specimens.

Systemic and genitourinary tract diseases change the volume, physical properties (e.g., pH, specific gravity, conductivity, color, turbidity, viscosity), suspended sediments (e.g., cells, minerals, casts), and other components of urine. The presence of excessive amounts of glucose and protein in urine, for example, invariably raises concerns about the presence of diabetes mellitus and/or renal pathology. These changes can be readily detected by routine urinalysis which includes assessment of the physical properties, sediments, cytology, and chemical composition (usually with a point-of-care dipstick “dry chemistry” analysis) (58, 59).

Over the past decade, “molecular urinalysis” involving urine metabolomics and urine biomarkers have been increasingly used in clinical research settings for disease detection. Mass spectrometry (MS), liquid/gas chromatography (LC/GC), nuclear magnetic resonance (NMR), capillary electrophoresis (CE), and kinetic nephelometry methods, have been used for detection of changes in urine analytes associated both with both normal physiology/metabolism and with disease states. These high technology approaches have had limited clinical translation. Magalhaes et al. (60) used CE/MS to identify polypeptide patterns in urine of human patients with kidney disease and diabetes. Patterns identified could not, however, be correlated with specific molecules (biomarkers) of physiological and pathophysiological significance. Darshi et al. (61) used MS to study diabetic kidney disease (DKD)-related metabolomic alterations. They noted their observations could not be readily applied to human patient management given the diverse spectrum of diabetes mellitus, chronic kidney disease (CKD), associated co-morbidities, and lack of correlation to standard metrics to ongoing, progressive disease trends, and patient demographics. In perspective: MS-based metabolomics are not used for routine patient care. The complexities of many systemic diseases, and the need for collection of large datasets to validate such technology-intensive methods, makes their use unlikely and cost-prohibitive—certainly out of the reach for most animal owners and their healthcare providers. The expense of purchasing and maintaining laboratory MS-based technologies, expertise required for operation and interpretation of results, lack of assay validation with large datasets of normal and abnormal specimens, will continue to limit clinical use.

### Rametrix^®^ molecular urinalysis

We recognized the limitations and challenges associated with MS-based metabolomics and biomarker assays. To address these, we invented and extensively validated a RS technology—Rametrix^®^ Molecular Urinalysis (RMU) (19, 20, 22, 24, 28, 30). The basic ideas behind RMU are demonstrated in Figures 1, 2 and involve additional ML/AI models to discover disease-specific spectral fingerprints in Raman spectra of urine. As previously stated, RMU technology, capitalizing on the many RS features previously described has these advantages over other molecular urinalysis technologies is rapid (<15 s per sample), inexpensive, requires small sample volumes (*ca.* 1.5 mL), is non-destructive of samples, requires no sample processing, can be done on thawed, frozen samples (which are stable for months), can be adapted to point-of-care testing and automation, and draws on data from our extensive RS databases.

We have applied RMU to study urine molecular composition of healthy human volunteers (27), human patients with CKD 4–5 (30), DKD (23), chronic Lyme disease (29), COVID19 (24, 26), and dogs with cancer (19), among other health and disease states. As one component of our recent study of dogs with cancer (unpublished data), we analyzed the urine of eleven (11) dogs with a variety of CNS neoplasms (above), with RMU. Although the number of patients with each tumor histotype was small, the spectral fingerprints of all tumors, considered as representative of brain tumors, was significantly different from healthy dogs (*n* = 89), and dogs with other common tumors, including lymphoma (*n* = 53), bladder cancer (*n* = 18), and mast cell tumors (*n* = 17). In perspective: it appears RMU can detect and differentiate canine tumors localized in the CNS.

## Discussion

In this Perspective, we have discussed the basics of RS and the application of this powerful technology to disease detection and management, including diseases of the CNS. There has been significant interest and research in applying RS to human neurodegenerative diseases and to detection and management of gliomas. We developed RMU as an RS-based platform and have used it to study both human and animal (canine) disease. We have validated the technology with studies in >4,000 human, >200 canine, and > 250 equine urine samples, collected for clinically-healthy individuals and from those with a variety of diseases. As a next phase of our work, we intend to focus on expanding our studies using RMU for detecting and as an adjunct to managing canine cancer. Specifically, we will determine how useful RMU is for pre-operative detection and post-operative surveillance of CNS malignancies and use it to discover spectral fingerprints other infectious, inflammatory, traumatic, and degenerative disease in companion animals.

## Data Availability

The data presented in the study are deposited in https://github.com/SengerLab/Raman-Scans/tree/Canine.
